# Supplementary material for: Ecotoxicology Evaluation of a Fenton—Type Process Catalyzed with Lamellar Structures Impregnated with Fe or Cu for the Removal of Amoxicillin and Glyphosate
Source: Int J Environ Res Public Health. 2023 Dec 13;20(24):7172. doi: 10.3390/ijerph20247172 (PMC10743043; doi:10.3390/ijerph20247172)
Supplement: Supplementary file 1 [file ijerph-20-07172-s001.zip › ijerph-2589085-supplementary.pdf]

International Journal of
Environmental Research and Public Health

MDPI

Suplementary material

Table S1: Results of the statistical analyzes carried out in the bioassays and the mutagenicity index are

- ***Lactuca sativa* amoxicillin (Amx)**

One-factor variance analysis

SUMMARY

Groups	*Account*	*Addition*	*Average*	*Variance*
treatment	5	15	3	2,5
100	5	174,9	34,98	256,60435

VARIANCE ANALYSIS

Origin of variations	*Sum of squares*	*Degrees of freedom*	*mean squares*	*F*	*Probability*	*Critical value for F*
Between groups	2556,801	1	2556,801	19,73568564	0,002160785	5,317655072
Within the groups	1036,4174	8	129,552175			
Total	3593,2184	9				

- ***Lactuca sativa* glyphosate (Gly)**

One-factor variance analysis

SUMMARY

Groups	*Account*	*Addition*	*Average*	*Variance*
treatment	5	15	3	2,5
100%	5	245,78	49,156	53,03273

VARIANCE ANALYSIS

Origin of variations	*Sum of squares*	*Degrees of freedom*	*mean squares*	*F*	*Probability*	*Critical value for F*
Between groups	5325,94084	1	5325,94084	191,8126784	7,14171E-07	5,317655072
Within the groups	222,13092	8	27,766365			
Total	5548,07176	9				

- ***Selenastrum capricornutum* amoxicillin (Amx)**

One-factor variance analysis

SUMMARY

Groups	*Account*	*Addition*	*Average*	*Variance*
treatment	5	15	3	2,5
10%	5	270	54	2008

VARIANCE ANALYSIS

Origin of variations	*Sum of squares*	*Degrees of freedom*	*mean squares*	*F*	*Probability*	*Critical value for F*
Between groups	6502,5	1	6502,5	6,468540164	0,034531176	5,317655072
Within the groups	8042	8	1005,25			
Total	14544,5	9				

- ***Selenastrum capricornutum* glyphosate (Gly)**

One-factor variance analysis

SUMMARY

Groups	*Account*	*Addition*	*Average*	*Variance*
treatment	5	15	3	2,5
10%	5	329	65,8	1044,2

VARIANCE ANALYSIS

Origin of variations	*Sum of squares*	*Degrees of freedom*	*mean squares*	*F*	*Probability*	*Critical value for F*
Between groups	9859,6	1	9859,6	18,83940002	0,002476927	5,317655072
Within the groups	4186,8	8	523,35			
Total	14046,4	9				

- ***Daphnia magna* amoxicillin (Amx)**

One-factor variance analysis

SUMMARY

Groups	*Account*	*Addition*	*Average*	*Variance*
treatment	5	15	3	2,5
25%	5	149	29,8	726,7

VARIANCE ANALYSIS

Origin of variations	*Sum of squares*	*Degrees of freedom*	*mean squares*	*F*	*Probability*	*Critical value for F*
Between groups	1795,6	1	1795,6	5,92484915	0,057253499	5,317655072
Within the groups	2916,8	8	364,6			
Total	4712,4	9				

- ***Daphnia magna* glyphosate (Gly)**

One-factor variance analysis

SUMMARY

Groups	*Account*	*Addition*	*Average*	*Variance*
treatment	5	15	3	2,5
25%	5	264	52,8	474,7

VARIANCE ANALYSIS

Origin of variations	*Sum of squares*	*Degrees of freedom*	*mean squares*	*F*	*Probability*	*Critical value for F*
Between groups	6200,1	1	6200,1	25,9853311	0,000932633	5,317655072
Within the groups	1908,8	8	238,6			
Total	8108,9	9				

- ***S. typhimurium* mutagenicity TA98 amoxicillin (Amx)**

One-factor variance analysis

SUMMARY

Groups	*Account*	*Addition*	*Average*	*Variance*
treatment	5	15	3	2,5
100%	5	6,6	1,32	0,317

VARIANCE ANALYSIS

Origin of variations	Sum of squares	Degrees of freedom	mean squares	F	Probability	Critical value for F
Between groups	7,056	1	7,056	5,009584665	0,05558045	5,317655072
Within the groups	11,268	8	1,4085			
Total	18,324	9				

- ***S. typhimurium*** **mutagenicity TA98 glyphosate (Gly)**

One-factor variance analysis

SUMMARY

Groups	Account	Addition	Average	Variance
treatment	5	15	3	2,5
100%	5	4,2	0,84	0,053

VARIANCE ANALYSIS

Origin of variations	Sum of squares	Degrees of freedom	mean squares	F	Probability	Critical value for F
Between groups	11,664	1	11,664	9,137485311	0,016488376	5,317655072
Within the groups	10,212	8	1,2765			
Total	21,876	9				

- ***S. typhimurium*** **mutagenicity TA100 amoxicillin (Amx)**

One-factor variance analysis

SUMMARY

Groups	Account	Addition	Average	Variance
treatment	5	15	3	2,5
100%	5	32,6	6,52	60,142

VARIANCE ANALYSIS

Origin of variations	Sum of squares	Degrees of freedom	mean squares	F	Probability	Critical value for F
Between groups	30,976	1	30,976	0,988985026	0,349114486	5,317655072
Within the groups	250,568	8	31,321			
Total	281,544	9				

- ***S. typhimurium* mutagenicity TA100 glyphosate (Gly)**

One-factor variance analysis

SUMMARY

Groups	*Account*	*Addition*	*Average*	*Variance*
treatment	5	15	3	2,5
100%	5	5,6	1,12	0,197

VARIANCE ANALYSIS

Origin of variations	*Sum of squares*	*Degrees of freedom*	*mean squares*	*F*	*Probability*	*Critical value for F*
Between groups	8,836	1	8,836	6,552465703	0,033658572	5,317655072
Within the groups	10,788	8	1,3485			
Total	19,624	9				
